# Diet quality and snack preferences of Turkish adolescents in private and public schools

**DOI:** 10.3389/fpubh.2024.1365355

**Published:** 2024-03-01

**Authors:** Fatma Elif Sezer, İdil Alpat Yavaş, Neda Saleki, Hande Bakırhan, Merve Pehlivan

**Affiliations:** ^1^Department of Nutrition and Dietetics, Institute of Health Sciences, Istanbul Medipol University, Istanbul, Türkiye; ^2^Department of Nutrition and Dietetics, Faculty of Health Sciences, Istanbul Medipol University, Istanbul, Türkiye; ^3^Department of Nutrition and Dietetics, Faculty of Health Sciences, Kahramanmaraş Istiklal University, Kahramanmaraş, Türkiye

**Keywords:** adolescent, diet quality, mean adequacy ratio, nutrient adequacy ratio, snack consumption, socioeconomic level

## Abstract

**Introduction:**

Socioeconomic level is one of the important factors determining diet quality. Snack preferences are affected by socioeconomic level. The objective of this research was to determine the effect of socioeconomic levels on diet quality and snack preferences among adolescents from different socioeconomic backgrounds.

**Methods:**

The study involved 118 adolescents aged between 10-18 years residing in Istanbul. A questionnaire prepared by the researchers was used to obtain information on the adolescents’ dietary habits, consumption of main meals and snacks, habits, and food consumption records. The participants’ food consumption was assessed using the retrospective 24-hour recall method, and diet quality was evaluated using the calculated nutrient adequacy ratio (NAR) and mean adequacy ratio (MAR).

**Results:**

The mean age of the adolescents was 16.42±0.89 years. The number of snacks consumed in private schools was found to be higher than in public schools (*p* < 0.05). The NAR score for vitamin C consumption was significantly higher in private schools compared to public schools (*p* < 0.05). Although the MAR scores of adolescents in private schools were higher than those in public schools, this difference was not statistically significant. The majority of adolescents in private schools regularly consumed fresh fruit (67.2%), milk (60.3%), yogurt (60.3%), and nuts (56.9%) as snacks. In contrast, 45% of adolescents in public schools regularly consumed pastries (*p* < 0.05).

**Discussion:**

It was observed that adolescents studying in public schools had a lower tendency to prefer healthy foods for snacks compared to those in private schools. Socioeconomic level was identified as an important factor influencing eating habits during adolescence. Considering that the level of income is significantly different between the adolescents studying at private and public schools, the higher consumption of snacks by the adolescents studying at private school may be associated with higher income.

## 1 Introduction

Nutrition is an important determinant in maintaining vital functions, supporting the immune system, ensuring growth and development, and maintaining a healthy life (1). It is important to adopt healthy lifestyle behaviors and a balanced diet to achieve a healthy and quality life throughout the life cycle. In order to achieve the desired quality of life, it is necessary to transform the healthy diet model into a lifestyle by increasing the awareness of nutrition throughout society (2).

In adolescence, a transition period from childhood to adulthood, growth and development accelerate, and cognitive and psychosocial development occur. Changes that occur during this period, affect the individual’s physical appearance, cognitive, and emotional development. A rapid increase in growth during adolescence results in an increase in the need for nutrients and energy, and nutrition is critically important in adolescence (3).

It is seen that adolescents who eat with their peers frequently prefer fast-food products that are high in energy, saturated fatty acids, sugar, and salt. Fast-food products are unhealthy options for adolescents who are in the growth and development period, as they are insufficient in many nutrients such as dietary fiber, vitamins A and C, and calcium. A diet pattern that is rich in energy and fat increases the risk of obesity in adolescents (4). When the diets of adolescents are examined in a study, it has been indicated that fat, saturated fat and sugar intakes were high, dairy products, fruit, and vegetable consumption were insufficient, and their diet quality was low (5). In a study, it has been observed that the measures of snacking were directly associated with higher energy, lower fruit/vegetable, higher sugar-sweetened beverages, and more frequent fast-food intakes (6). Poor diet quality may lead to several chronic diseases including diabetes, heart disease, stroke, cancer, and obesity and also negatively affects growth and development and school success (7). Regular meal consumption, healthy food choices in meals and energy, and macro and micronutrient intake in the diet as recommended amounts affect the health of adolescents positively. For healthy adulthood, it is of great importance to acquire healthy eating habits in childhood and adolescence (3).

Psychosocial, environmental, and sociodemographic factors also play an important role in the food choices of adolescents. The food choices of parents, which vary depending on sociodemographic characteristics, are also reported as an important factor that determines the nutritional habits of individuals at this age (8). In addition, it is emphasized that nutritional problems seen in childhood and adolescence are closely related to many sectors such as the economy and education (9). Researchers have found that socioeconomic factors, such as living in smaller houses, having a low socioeconomic level, having many family members, and having a low monthly income, are significantly associated with the food choices and diet quality of adolescents (10). In a study conducted with university students, the researchers found that students with low socioeconomic status consumed unhealthy foods significantly more than their peers with higher socioeconomic status (11). In another study conducted with 1,000 adolescents, it has been observed that adolescents living in regions with low socioeconomic status showed higher fast food consumption (12). In studies conducted in different countries, it has been shown that especially adolescents and young adults with low socioeconomic level consume excessive calories, and the researchers emphasized that socioeconomic level can directly affect eating behavior (13, 14).

There are studies investigating the nutritional status of adolescents in Turkey, but studies on snack patterns, snack preferences, and diet quality are limited (15–18). Scarce research exists that contrasts the snacking habits and diet quality of students enrolled in private and public schools. To the best of our knowledge, this research constitutes the first comprehensive examination of adolescent snack preferences within the context of socioeconomic standing in Turkey. This study aims to determine the effects of socioeconomic status on adolescents’ snack patterns, snack preferences, and diet quality.

## 2 Materials and methods

### 2.1 Research location, time, and sample selection

**Figure 1:**
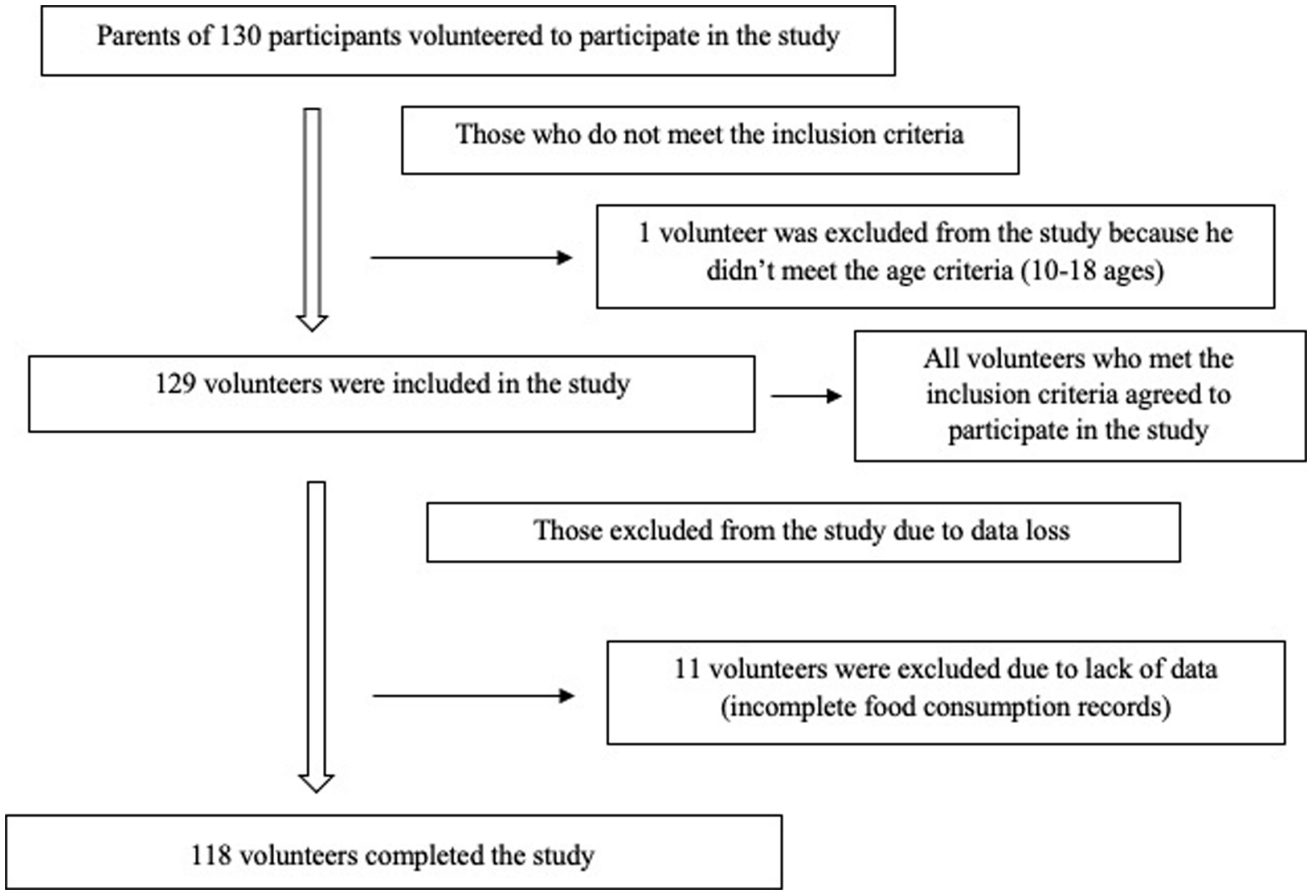
Participants recruitment flow chart.

The research was conducted on randomly selected adolescents aged 10–18 years who applied to the Nutrition and Diet Polyclinic at Istanbul Medipol University between January and June 2020. The study was conducted with a total of 118 high school students enrolled in public schools (*n* = 60) and private schools (*n* = 58) living in Istanbul (Figure 1). Parental consent was obtained for all children and adolescents included in this study.

The G*Power 3.1 software was used to calculate the sample size, and it was determined that 51 participants needed to be included in each group with an effect size of 0.5 and a power of 0.8. In this study, a significance level of *p* < 0.05 was accepted.

In this cross-sectional study, “Ethics Committee Approval” was obtained from Istanbul Medipol University Non-Interventional Clinical Research Ethics Committee with the number E-10840098-772.02-6957 and dated November 15, 2022. Voluntary information and consent forms were obtained from the adolescents who agreed to participate in the study. This study was conducted in accordance with the principles of the Declaration of Helsinki (“World Medical Association Declaration of Helsinki: Ethical Principles for Medical Research Involving Human Subjects,” 2014).

### 2.2 Data collection and evaluation

To collect the research data, a questionnaire form created by the researcher was used, and all data were collected using a face-to-face interview method. The questionnaire form has 4 parts and sociodemographic data, anthropometric measurements, nutritional behaviors, and snack and food consumption of participants were collected.

The minimum wage levels in the year the research was conducted were 2000 TL. Therefore, income grouping is planned as >2000 TL and its multiples. Income levels are categorized as very low, low, medium, high, and very high, according to minimum wage (19).

During the anthropometric measurements of the participants, their height was measured using a wall-mounted height measuring tape, and their body weight was measured using a digital scale. Body mass index (BMI) was calculated by dividing the body weight by the height squared (kg/m^2^). An evaluation was made according to percentile curves suitable for the age and gender of the adolescents. Percentile values and curves developed for Turkish children by Neyzi et al. (20) were used, and percentile values were classified as thin (<5), normal (5–85), overweight (85–95), and obese (≥95).

The number of daily meals, whether they skip meals, where they eat their meals, the opportunity of snacks during the time spent at school, the type of foods they prefer for snacks, and the frequency of snack consumption were questioned. Food and beverages are grouped as milk, yogurt, ayran, kefir, cheese, fresh fruit, dried fruit, salad, nuts, pastries, rusk, biscuits, crackers, chips, tea, coffee, fruit juice, and soft drinks. Adolescents were classified as regular consumers, if they consumed these snacks 4 or more times a week, and as occasional consumers if they consumed less than 4 times a week (21).

In the final part of the questionnaire, the participant’s 24-h food consumption was assessed using the retrospective recall method. Participants were asked to specify all the foods and beverages they consumed over the previous 24 h, along with the amounts they consumed, using measures such as tablespoons, glasses, bowls, slices, or weight. Dietary recall data were collected by the researcher guiding the participants to accurately describe their food consumption, using the “Food and Food Photo Catalog” (22). The researcher evaluated the data obtained from the food consumption record, using the Nutrition Information Systems Package Program (BeBiS), which is a software program that analyzes the nutrient content of foods.

The nutrient adequacy ratio (NAR) and mean adequacy ratio (MAR) were calculated using the data from the food consumption records that were analyzed using BeBiS to determine the diet quality of the participants and to evaluate their adequate intake of some nutrients. The nutrients used in the calculation are carbohydrates, protein, calcium, iron, magnesium, phosphorus, folate, vitamin B12, riboflavin, and niacin. If the NAR score is above 1, it is considered to meet the recommended intake. NAR was calculated using the following formula (23).

NAR = Daily intake of the nutrient/Recommended daily intake of the nutrient.

The dietary reference intakes (DRI) specified by the National Institutes of Health (NIH) were used to determine the DRIs for the nutrients used in the NAR calculation (24). After the NAR was calculated, the MAR calculation was made using the following formula (23).

MAR = Sum of all NAR rates/Total number of nutrients.

### 2.3 Statistical analysis

IBM SPSS Statistics 21.0 was used to analyze the data (25). The acceptable error rate was 5% with a confidence level of 95%. Percentages were used for qualitative variables and mean and standard deviations were used for quantitative parametric variables. The normality of distribution was tested with the Kolmogorov–Smirnov test. The significance of the intergroup distribution of descriptive statistics was tested by Fisher’s Chi-square test. Kruskal–Wallis test and Mann–Whitney *U*-test were applied to data that was not normally distributed.

## 3 Results

This study was conducted with a total of 118 adolescents (48.3% boys and 51.7% girls) with a mean age of 16.4 ± 0.89 years. The demographic characteristics and anthropometric measurements of adolescents are given in Table 1. The income level of the majority of the adolescents studying in the private school was good (34.5%) and very good (50.0%), while those studying in the public school were medium or worse (63.3%). The educational status of the parents of adolescents in the private school was significantly higher than those in the public school (*p* > 0.05).

**Table 1:** Demographic characteristics and anthropometric measurements of adolescents.

	Private school (*n* = 58)		Public school (*n* = 60)		Total (*n* = 118)		*p*
	*n*	%	*n*	%	*n*	%	
*Gender*							
Male	25	43.1	32	53.3	57	48.3	
Female	33	56.9	28	46.7	61	51.7	
Age (years) (Mean ± SD)	16.0 ± 0.91		16.8 ± 0.68		16.4 ± 0.89		
*Level of income*							
Very low	–	–	3	5.0	3	2.5	
Low	1	1.7	11	18.3	12	10.2	
Medium	8	13.8	24	40.0	32	27.1	**<0.001** ^*^
High	20	34.5	14	23.4	34	28.8	
Very high	29	50.0	8	13.3	37	31.4	
*Mother’s education level*							
Primary school or lower	3	5.2	19	31.7	22	18.6	**<0.001** ^*^
Middle school, high school	26	44.8	26	43.3	52	44.1	
College or higher	29	50.0	15	25.0	44	37.3	
*Father’s education level*							
Primary school or lower	–	–	15	25.0	15	12.7	
Middle school, high school	22	37.9	29	48.3	51	43.2	**<0.001** ^*^
College or higher	36	62.1	16	26.7	52	44.1	
Number of people living in the family (Mean ± SD)	3.5 ± 0.77		4.2 ± 0.99		3.9 ± 0.94		**<0.001** ^*^
*Anthropometric measurements*							
Height (cm) (X̅ ±SD)	171.4 ± 7.76		172.4 ± 9.62		171.9 ± 8.73		0.543
Body weight (kg) (X̅ ±SD)	65.3 ± 13.63		64.6 ± 13.89		65.0 ± 13.70		0.792
BMI (kg/m^2^) (X̅ ±SD)	22.2 ± 3.80		21.5 ± 3.40		21.9 ± 3.61		0.288

Categorization of BMI	*n*	%	*n*	%	*n*	%	
Underweight	9	15.5	11	18.3	20	16.9	0.605
Normal weight	34	58.7	38	63.4	72	61.0	
Overweight	14	24.1	8	13.3	22	18.6	
Obese	1	1.7	3	5.0	4	3.5	

^*^Fisher’s Chi-square test, *p* < 0.05.

Table 2 shows the frequency of consumption of meals and snacks and the places where adolescents consume these meals. It was observed that the majority of adolescents studying at private schools (81.0%) and public schools (81.7%) consumed 3 or more main meals a day (*p* > 0.05). Considering their snack consumption, 74.1% of the adolescents studying at private schools consume 2 or more snacks a day, while 80% of those in public schools have 1 or 2 snacks, and this difference is statistically significant (*p* < 0.05). While the majority of the adolescents studying in private school consume breakfast and dinner at home (84.5 and 91.4%, respectively), approximately half of them consume their lunch in the school cafeteria (48.3%), the adolescents in public school consume all three main meals (breakfast 73.3%, lunch 81.7% and dinner 75.0%) in the school cafeteria (*p* < 0.05).

**Table 2:** Meal frequency of adolescents and places they consume their meals.

	Private school (*n* = 58)		Public school (*n* = 60)		
	*n*	%	*n*	%	*p*
*Number of meals per day*					
1	–	–	1	1.6	0.905
2	11	19.0	10	16.7	
3 or more	47	81.0	49	81.7	
*Number of snacks per day*					
1	15	25.9	30	50.0	**0.02** ^*^
2	22	37.9	18	30.0	
3 or more	21	36.2	12	20.0	
**Places adolescents consume their meals**					
*Breakfast*					
Home	49	84.5	15	25.0	**0.000** ^*^
School cafeteria	–	–	44	73.3	
School canteen	9	15.5	1	1.7	
*Lunch*					
Home	10	17.2	6	10.0	**0.001** ^*^
School cafeteria	28	48.3	49	81.7	
School canteen	3	5.2	2	3.3	
Restaurant/cafe/peddler	17	29.3	3	5.0	
*Dinner*					
Home	53	91.4	14	23.3	**<0.001** ^*^
School cafeteria	–	–	45	75.0	
Restaurant/cafe	5	8.6	1	1.7	
*Snack*					
Home	27	46.6	3	5.0	**<0.001** ^*^
School canteen, market	31	53.4	47	78.4	
School provides snacks	–	–	2	3.3	
Dormitory cafeteria	–	–	8	13.3	

^*^Fisher’s Chi-square test, *p* < 0.05.

The factors that affect the snack preferences of adolescents in the study are given in Table 3. While the most important factors that affect snack preferences are hygiene (58.9%) and food being healthy (66.1%) for students in private school, easy access (58.4%) and price (64.9%) were more important for students in public school (*p* < 0.05).

**Table 3:** Distribution of factors affecting adolescents’ snack preferences.

		Private school		Public school		
		*n*	%	*n*	%	*p*
Healthy	Yes	41	66.1	21	33.9	**<0.001** ^*^
	No	17	30.4	39	69.6	
Easily accessible	Yes	37	41.6	52	58.4	**0.004** ^*^
	No	21	72.4	8	27.6	
Price	Yes	26	35.1	48	64.9	**<0.001** ^*^
	No	32	72.7	12	27.3	
Hygiene	Yes	43	58.9	30	41.1	**0.007** ^*^
	No	15	33.3	30	66.7	
Habits	Yes	46	52.3	42	47.7	0.246
	No	12	40.0	18	60.0	
Taste	Yes	54	51.4	51	48.6	0.160
	No	4	30.8	9	69.2	

^*^Chi-square test, *p* < 0.05.

Data on the diet quality of adolescents are shown in Table 4. Adolescents in private school had higher NAR scores for vitamin B2 (0.85 ± 0.43), folate (0.47 ± 0.29), potassium (0.62 ± 0.30), calcium (0.39 ± 0.20) and magnesium (0.49 ± 0.21) than adolescents in public school. However, this difference was not statistically significant (*p* > 0.05). The NAR score for vitamin C was statistically significantly higher in adolescents in private school (0.93 ± 0.89) than in adolescents in public school (0.44 ± 0.34) (*p* > 0.05).

**Table 4:** Evaluation of diet quality of adolescents.

	Private school	Public school	
	Mean ± SS	Mean ± SS	*p*
*NAR*			
Protein	0.99 ± 0.34	1.01 ± 0.34	0.751
Dietary fiber	0.47 ± 0.26	0.48 ± 0.19	0.541
Vitamin B_2_	0.85 ± 0.43	0.77 ± 0.38	0.379
Folate	0.47 ± 0.29	0.43 ± 0.17	0.910
Vitamin C	0.93 ± 0.89	0.44 ± 0.34	**<0.001** ^*^
Potassium	0.62 ± 0.30	0.60 ± 0.24	0.884
Calcium	0.39 ± 0.20	0.35 ± 0.17	0.392
Magnesium	0.49 ± 0.21	0.48 ± 0.16	0.822
Iron	0.57 ± 0.31	0.59 ± 0.21	0.230
Zinc	0.69 ± 0.28	0.75 ± 0.31	0.245
Niacin	0.59 ± 0.29	0.59 ± 0.20	0.962
MAR	0.64 ± 0.28	0.59 ± 0.20	0.256

^*^Mann–Whitney *U*-test, *p* < 0.05.

The distribution of the frequency of consumption of the foods that adolescents prefer for snacks is given in Table 5. It has been determined that the majority of adolescents studying in private school regularly consume fresh fruit (67.2%), milk (60.3%), yoğurt (60.3%), and nuts (56.9%) as a snack, whereas most of the adolescents studying in public school do not consume milk (41.7%), yogurt (60.0%), ayran (55.0%), and cheese (68.3%) at all. While 45% of the adolescents studying in public school stated that they regularly consume pastries, this rate is 17.2% in private school (*p* < 0.05). A statistically significant difference was found between adolescents studying in private and public schools in terms of consumption of milk and dairy products, fresh and dried fruits, nuts, and pastries in snacks (*p* < 0.05).

**Table 5:** Snack preferences of adolescents.

	Private school			Public school			
	Regular consumer	Occasional consumer	Never consumer	Regular consumer	Occasional consumer	Never consumer	
Besinler	*n* (%)	*n* (%)	*n* (%)	*n* (%)	*n* (%)	*n* (%)	*p*
Milk	35 (60.3)	15 (25.9)	8 (13.8)	13 (21.7)	22 (36.6)	25 (41.7)	**<0.001** ^*^
Yogurt	35 (60.3)	16 (27.6)	7 (12.1)	5 (8.3)	19 (31.7)	36 (60.0)	**<0.001** ^*^
Ayran	23 (39.7)	26 (44.8)	9 (15.5)	18 (30.0)	9 (15.0)	33 (55.0)	**<0.001** ^*^
Kefir	7 (12.1)	10 (17.2)	41 (70.7)	2 (3.3)	3 (5.0)	55 (91.7)	**0.014** ^*^
Cheese	22 (37.9)	15 (25.9)	21 (36.2)	9 (15.0)	10 (16.7)	41 (68.3)	**0.002** ^*^
Fresh fruit	39 (67.2)	14 (24.1)	5 (8.7)	15 (25.0)	24 (40.0)	21 (35.0)	**<0.001** ^*^
Dried fruit	19 (32.8)	31 (53.4)	8 (13.8)	4 (67)	22 (36.6)	34 (56.7)	**<0.001** ^*^
Salad	29 (50.0)	16 (27.6)	13 (22.4)	5 (8.3)	18 (30.0)	37 (61.7)	**<0.001** ^*^
Nuts	33 (56.9)	22 (37.9)	3 (5.2)	13 (21.7)	22 (36.6)	25 (41.7)	**<0.001** ^*^
Pastries	10 (17.2)	32 (55.2)	16 (27.6)	27 (45.0)	26 (43.3)	7 (11.7)	**0.003** ^*^
Breadsticks	7 (12.1)	23 (39.6)	28 (48.3)	3 (5.0)	8 (13.3)	49 (81.7)	**0.001** ^*^
Biscuit	18 (31.0)	28 (48.3)	12 (20.7)	26 (43.3)	26 (43.3)	8 (13.3)	0.317
Crackers	14 (24.1)	28 (48.3)	16 (27.6)	20 (33.3)	22 (36.7)	18 (30.0)	0.394
Chips	6 (10.3)	24 (41.4)	28 (48.3)	9 (15.0)	24 (40.0)	27 (45.0)	0.747
Tea	44 (75.8)	7 (12.1)	7 (12.1)	48 (80.0)	4 (6.7)	8 (13.3)	0.599
Coffee	39 (67.2)	13 (22.4)	6 (10.4)	31 (51.7)	20 (33.3)	9 (15.0)	0.227
Fruit juice	9 (15.5)	27 (46.6)	22 (37.9)	5 (8.3)	32 (53.4)	23 (38.3)	0.459
Soft drinks	10 (17.2)	21 (36.2)	27 (46.6)	8 (13.3)	24 (40.0)	28 (46.7)	0.816

^*^Fisher’s Chi-square test, *p* < 0.05.

## 4 Discussion

In this study, it was aimed to evaluate the diet quality and nutritional status of adolescents studying in private and public schools, and to compare their snack preferences and consumption frequencies in a sample reflecting different socioeconomic levels.

Studies conducted in different provinces of Turkey indicate that families with high monthly incomes more often prefer private schools. Additionally, as the total income of the family and the education level of the parents increase, so do education expenditures (26). In different studies conducted with adolescents, it has been reported that the type of school (public or private) that adolescents attend is accepted as a factor representing their socioeconomic status (27). Supporting the results in the literature, this study determined that the income status and parental education levels of the adolescents who are educated in public schools were statistically significantly lower than their peers who are educated in private schools.

The average height, body weight, and BMI of the adolescents included in this study are similar to the anthropometric measurements found in studies, conducted with adolescents in different provinces of Turkey (28, 29). When the BMI value specified by the Turkish Dietary Guidelines (TUBER) for the age of 16 is taken as a reference, the mean BMI of the adolescents included in the current study, whose mean age is 16.4 ± 0.89 years, is seen to be in the normal range (1). In a study conducted by Coşkun and Karagöz (29) with 220 adolescents, it was reported that 6.6% of adolescents were overweight and 4.2% were obese. Of the adolescents included in this study, 18.6% were overweight and 3.4% were obese. Although obesity rates are similar to those in other studies, the rate of obese adolescents is higher in this study. During the transition period between childhood and adolescence, a change in diet composition is observed, including higher consumption of snacks and soft drinks and lower intake of fruits and vegetables. This is thought to increase the rate of obesity and overweight (30).

Socioeconomic status is a strong determinant of body weight, obesity risk, and eating behaviors. The probability of developing obesity is higher in children from low-income families than in children from high-income families (30). However, this study found no statistically significant difference in anthropometric measurements between private school and public school adolescents. Supporting the findings of this study, a study reported that the prevalence of obesity was similar among adolescents from low and middle socioeconomic status and among adolescents from low and high socioeconomic status (31). It is not possible to draw a clear conclusion since obesity reflects the complex interactions between genetic, metabolic, behavioral, cultural, and environmental factors (32). More research is needed to help understand the underlying causes of obesity.

The number of meals per day varies according to the socioeconomic status of the family. In a study conducted with adolescents (*n* = 891), it was determined that adolescents with high socioeconomic status (72.3%) consumed 3 or more main meals at a higher rate compared to their peers with medium (61.8%) and low (54.8%) socioeconomic status (33). Another study reported that adolescents with higher socioeconomic status consumed more snacks than adolescents with lower socioeconomic status (34). In this study, it was found that the majority of adolescents consumed 3 or more main meals a day, and there was no statistically significant difference in terms of socioeconomic level. However, the number of snacks consumed daily was found to be significantly higher in adolescents studying at private schools. Considering that the level of income is significantly different between the adolescents studying at private and public schools, the higher consumption of snacks by the adolescents studying at private school may be associated with higher income.

It was observed that the rate of consuming breakfast and lunch at school was higher in adolescents who were educated in public schools and were offered free or affordable breakfast and lunch in their school (35). In a study conducted with adolescents aged 14–19 years studying in a public school in Brazil, it was found that the majority of adolescents (92.2%) ate lunch at home and only 4.9% ate at school (36). In the present study, it was observed that the majority of adolescents in public school consumed breakfast, lunch, and dinner in the school cafeteria. The fact that meals are usually consumed at school, especially in public schools, may be associated with a lower socioeconomic level. It is thought that food practices in schools aim to support the development of children and to give them healthy eating habits, especially children from low-income families.

In a study conducted with 166 adolescents aged 11–13 years in the United States, the factors that affect snack preferences were examined. The most important factor was found to be price, followed by nutritiveness, taste, and easy accessibility (37). In another study of children aged between 7 and 12, it was found that availability and price influenced children’s food choices and purchasing decisions. The most commonly purchased foods (42% salty packaged snacks) were the most common and least expensive ones in grocery stores around public schools (38). In this study, the most important factors that affected the preference of snacks for private school students were hygiene and health, and easy accessibility and price for students in public school. Given that the majority of students in public school are from middle- and lower-income families, pocket money is likely to be limited. For this reason, the price factor was found to be statistically significantly more important in snack preferences according to socioeconomic level. This result is similar to the findings of other studies (37, 39, 40).

Diet quality indices, such as NAR and MAR scores, compare an individual’s nutrient intake with age- and gender-specific recommended intake levels to assess the quality of the diet (40). In the Study of Cardiovascular Risks in Adolescents (ERICA), in which the diet quality of 71,553 adolescents was evaluated by Ronca et al., adolescents were found to have poor diet quality (41). In middle school children, considering that NAR scores of vitamin C (boys and girls 0.4 ± 0.3), calcium (boys 0.4 ± 0.3 and girls 0.4 ± 0.2), iron (boys 0.8 ± 0.3, girls 0.7 ± 0.3), vitamin A (boys 0.8 ± 0.4, girls 0.9 ± 0.4), and folic acid (boys 0.9 ± 0.5, girls 0.8 ± 0.4) were low, it is observed that there was insufficient intake of these nutrients. It was observed that the greatest deficiency in both genders was calcium and vitamin C intake, followed by vitamin A, iron, and folic acid. Mean MAR scores were reported to be low (0.9 ± 0.3) in both genders (42). Higher socioeconomic status is associated with better diet quality. Studies indicate that adolescents with high family income have a relatively higher intake of vitamins and minerals, which indicates better diet quality (43). In this study, diet quality was evaluated, and it was found that the MAR scores of the adolescents in the private school were higher than the students in the public school, but this difference was not statistically significant. Only vitamin C has a statistically significantly higher NAR score. It is thought that this may be due to the higher rate of regular consumption of fresh fruit and salad in private school adolescents (44, 45).

In a study, it was found that weekly consumption of soft drinks (51.7%) and sweets (44.1%) in children from families with low socioeconomic status was higher than that of children from families with high socioeconomic status (soft drinks 21.6%, sweets 24.9%), and it was stated that high socioeconomic status was associated with less consumption of energy-dense foods in children (46). In the study conducted by Moitra and Madan (2021) with adolescents (*n* = 712), it was reported that adolescents studying in private school (1.4 days/week) consumed cake and pastry as snacks more than adolescents in public school (0.7 days/week), and the consumption of biscuits and cookies was higher in adolescents studying in public school (6.9 days/week and 4.5 days/week, respectively) (47). According to the results of the Brazilian National Survey of School Health (PeNSE)-2015, the consumption of vegetables in adolescents aged 11–19 was higher in private school students (42.8%) than in public school students (36.8%), in the other hand, it has been reported that ultra-processed salty foods such as processed meats, packaged salty snacks, and crackers, which are expressed as unhealthy diet indicators, are consumed more in private school students (44). Schools should be prohibited from selling deep-fried foods and sugar-rich soft drinks. Instead, the canteens should be encouraged to sell healthy food and drinks, such as fruits and vegetables (48).

In this study, similar to Moitra and Madan’s study, consumption of pastries as snacks was found to be higher in adolescents with low socioeconomic status, and similar to the PeNSE-2015 report, salad and vegetable consumption was found to be higher in adolescents studying in private schools. However, unlike the PeNSE-2015 report, this study reported that the consumption of healthy snacks such as milk, fresh and dried fruits, and nuts, instead of highly processed and packaged snacks, was higher in adolescents studying in private schools.

### 4.1 Limitations of the study

The present study is important in terms of showing the snack preferences of adolescents in Turkey and the factors that affect their snack preferences. Personal awareness of appropriate nutrition is important in reducing the consumption of fast food and snack-type foods and in adopting healthy diets, and individuals could get away from overnutrition or malnutrition in accordance with their level of knowledge and socioeconomic level. Many factors influence dietary choices, but the environment and socioeconomic level are crucial components pointing these decisions. Unless individuals, and especially children, learn how to make appropriate food choices according to socioeconomic levels, they cannot avoid the negative influence of their social environment.

However, this study also has some limitations. The sample size of current study was small, and physical activity, regional differences, and other environmental factors could not be investigated. It should be kept in mind that the food consumption record covers only a single day retrospectively, and this may be insufficient to reflect the overall eating habits of that person.

### 4.2 Areas for further research

This study can be rescheduled to include a larger sample size and other environmental factors. The quality of the meals offered to students at private and public schools may be examined to determine the dietary quality of students. Adolescents should be educated about adequate and balanced nutrition so that they can make healthy choices from the food available in school canteens, and healthy foods that meet the daily energy and nutritional needs of students should be made available in school canteens and cafeterias. The age group, region, sample, and results of the present study can not be generalized to the rest of Turkey. Therefore, future research should involve a larger geographic region and sample size.

## 5 Conclusion

Since the nutritional habits acquired during adolescence lay the foundation for adult eating habits, it is of great importance to choose healthy foods and gain healthy eating habits during adolescence. Level of income can affect students’ snack preferences. In this study, the diet quality of adolescents was found to be similar, but socioeconomic status affects the factors that influence their snack preferences and the foods they prefer. The majority of children from high-income families who attend private schools, consider factors such as whether the food is healthy, hygienic, and delicious. However, it has been observed that children in public schools from low-income families prefer snacks based on their price and accessibility. Furthermore, it has been shown that children in private schools consume more healthy foods on a regular basis such as milk, yogurt, ayran, kefir, cheese and its varieties, dried fruit, salad, and nuts compared to children in public schools. These findings offer a deeper understanding of the role of socioeconomic level on snack preferences and diet quality of adolescents.

## Data availability statement

The raw data supporting the conclusions of this article will be made available by the authors, without undue reservation.

## Ethics statement

The studies involving humans were approved by Istanbul Medipol University Non-Interventional Clinical Research Ethics Committee. The studies were conducted in accordance with the local legislation and institutional requirements. Written informed consent for participation in this study was provided by the participants’ legal guardians/next of kin.

## Author contributions

FS: Writing – original draft, Writing – review & editing. İY: Writing – original draft, Writing – review & editing. NS: Writing – review & editing. HB: Writing – review & editing. MP: Writing – review & editing.
